# *catena*-Poly[[bis­[[bis­(3-amino­prop­yl)amine-κ^3^ *N*,*N*′,*N*′′](thio­cyanato-κ*N*)cadmium]-μ_4_-sulfato-κ^4^ *O*,*O*:*O*′,*O*′] methanol hemisolvate]

**DOI:** 10.1107/S1600536811031163

**Published:** 2011-08-06

**Authors:** Jan Boeckmann, Christian Näther

**Affiliations:** a Institut für Anorganische Chemie, Christian-Albrechts-Universität Kiel, Max-Eyth Strasse 2, D-24098 Kiel, Germany

## Abstract

The asymmetric unit of the title compound, {[Cd_2_(NCS)_2_(SO_4_)(C_6_H_17_N_3_)_2_]·0.5CH_3_OH}_*n*_, consists of two Cd^2+^ cations, two thio­cyanate and one sulfate anion, two bis­(3-amino­prop­yl)amine co-ligands and one methanol molecule with half-occupancy. Each Cd^2+^ cation is coordinated by four N atoms of one terminal *N*-bonded thio­cyanate anion and one bis­(3-amino­prop­yl)amine co-ligand, and by two O atoms of two symmetry-related sulfate anions, defining a slightly distorted octa­hedral coordination polyhedron. Each two Cd^2+^ cations are connected into dimers, which are located on centres of inversion and which are further μ-1,1:3,3-bridged *via* the sulfate anions into polymeric zigzag chains along the *a* axis.

## Related literature

For background information about thermal decomposition reactions and the resulting inter­mediates, see: Boeckmann & Näther (2010 ▶, 2011 ▶); Boeckmann *et al.* (2011 ▶); Wöhlert *et al.* (2011 ▶); Wriedt *et al.* (2009*a* ▶,*b* ▶); Wriedt & Näther (2010 ▶). 
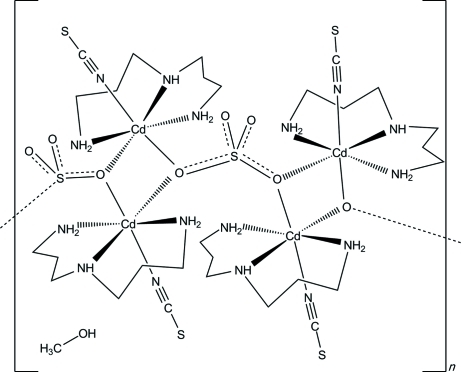


## Experimental

### 

#### Crystal data

[Cd_2_(NCS)_2_(SO_4_)(C_6_H_17_N_3_)_2_]·0.5CH_4_O
*M* *_r_* = 715.49
Triclinic, 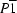

*a* = 10.6648 (9) Å
*b* = 12.4441 (12) Å
*c* = 12.9240 (12) Å
α = 61.359 (10)°
β = 69.064 (10)°
γ = 68.772 (10)°
*V* = 1367.3 (2) Å^3^
*Z* = 2
Mo *K*α radiation
μ = 1.82 mm^−1^
*T* = 200 K
0.18 × 0.13 × 0.09 mm

#### Data collection

STOE IPDS-1 diffractometer
Absorption correction: numerical (*X-SHAPE* and *X-RED32*; Stoe & Cie, 2008 ▶) *T*_min_ = 0.746, *T*_max_ = 0.841
12730 measured reflections
5736 independent reflections
4365 reflections with *I* > 2σ(*I*)
*R*_int_ = 0.040

#### Refinement

*R*[*F* ^2^ > 2σ(*F* ^2^)] = 0.042
*wR*(*F* ^2^) = 0.113
*S* = 1.07
5736 reflections
301 parameters
H-atom parameters constrained
Δρ_max_ = 1.07 e Å^−3^
Δρ_min_ = −0.87 e Å^−3^

### 

Data collection: *X-AREA* (Stoe & Cie, 2008 ▶); cell refinement: *X-AREA*; data reduction: *X-AREA*; program(s) used to solve structure: *SHELXS97* (Sheldrick, 2008 ▶); program(s) used to refine structure: *SHELXL97* (Sheldrick, 2008 ▶); molecular graphics: *XP* in *SHELXTL* (Sheldrick, 2008 ▶) and *DIAMOND* (Brandenburg, 2011 ▶); software used to prepare material for publication: *SHELXL97*.

## Supplementary Material

Crystal structure: contains datablock(s) I, global. DOI: 10.1107/S1600536811031163/bt5596sup1.cif

Structure factors: contains datablock(s) I. DOI: 10.1107/S1600536811031163/bt5596Isup2.hkl

Additional supplementary materials:  crystallographic information; 3D view; checkCIF report

## Figures and Tables

**Table 1:** Selected bond lengths (Å)

Cd1—N11	2.248 (4)
Cd1—N13	2.250 (5)
Cd1—N1	2.347 (5)
Cd1—N12	2.351 (5)
Cd1—O1^i^	2.388 (3)
Cd1—O1	2.619 (3)
Cd2—N23	2.247 (5)
Cd2—N21	2.250 (5)
Cd2—N2	2.283 (6)
Cd2—N22	2.374 (4)
Cd2—O3	2.386 (3)
Cd2—O3^ii^	2.676 (4)

Symmetry codes: (i) 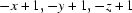; (ii) 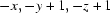.

**Fig. 1.**
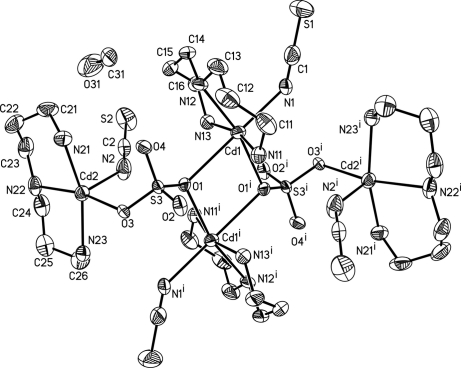
: Crystal structure of the title compound, showing the coordination around the Cd2+ cations with labelling and displacement ellipsoids drawn at the 50% probability level. The H-atoms are omitted for clarity.

**Fig. 2.**
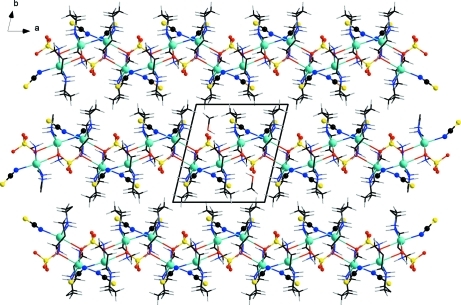
: Packing diagram of the title compound with view along the crystallographic c axis (aqua = cadmium; yellow = sulfur; red = oxygen; blue = nitrogen; black = carbon; light-grey = hydrogen).

## supplementary crystallographic information

### Comment

Recently, we reported about the thermal decomposition reaction as a tool for the selective synthesis of one-dimensional and two-dimensional coordination compounds [Boeckmann & Näther (2010); Boeckmann & Näther (2011); Boeckmann *et al.* (2011); Wöhlert *et al.* (2011); Wriedt *et al.* (2009*a*,*b*); Wriedt & Näther (2010)]. In this approach precursors based on paramagnetic transition metal thio- and selenocyanates and bidentate and monodentate *N*-donor co-ligands are heated leading to a stepwise loss of the neutral co-ligands and the formation of higher condensed networks with modified magnetic exchange interactions. Unfortunately, structure determination of the ligand-deficient intermediates is very often difficult to achieve because most of them can only prepared by thermal decomposition which leads to powders of very poor crystallinity. This problem can be overcome by preparing similar compounds based on cadmium(II) thio- and selenocyanates, which can easily be crystallized from solution and which are very often isotypic to their paramagnetic counterparts. In this case the structures of the paramagnetic intermediates can simply be determined using the Rietveld method. In this connection, we tried to prepare a ligand-deficient intermediate on the basis of Cd(NCS)_2_ and the tridentate co-ligand bis(3-aminopropyl)amine. Surprisingly a mixed anionic chain structure was obtained which was characterized by single crystal X-ray diffraction.

In the crystal structure of the title compound the cadmium(II) cations are coordinated by four nitrogen atoms of one terminal *N*-bonded thiocyanato anion, one tridentate co-ligand bis(3-aminopropyl)amine and two oxygen atoms of two symmetry related sulfate anions within a slightly distorted octahedral coordination geometry (Fig.1 and Tab.1). One of the Cd-O distances to Cd2 of 2.676 (4) Å is slightly elongated. These octahedra are bridged *via* the sulfur oxygen atoms into dimeric Cd^2+^ units that are located on centers of inversion. These units are further connected into zigzag chains that elongate in the direction of the crystallographic *a* axis (Fig.2).

### Experimental

The title compound was prepared by the reaction of 128.2 mg CdSO_4_^.^8/3H_2_O (0.50 mmol), 153.8 mg Ba(NCS)_2_^.^3H_2_O (0.50 mmol) and 35.2 µ*L* bis(3-aminopropyl)amine (0.25 mmol) in 1.50 ml methanol at RT in a closed 3 ml snap cap vial. After two days colourless blocks of the title compound were obtained.

### Refinement

The position of the methanol molecule seems to be occupied to only 50%. If full occupation is assumed, unusual large anisotropic displacement parameters and higher R values are obtained. If an s.o.f. of 0.5 is used, all reliabilty factors drop down, the anisotropic displacement ellipsoids looks reasonable and no residual electron density indicating disorder is found.

All H atoms were were positioned with idealized geometry (O-H allowed to rotate but not to tip) and were refined using a riding model with *U*_eq_(H) = 1.2 *U*_eq_(C,N) or *U*_eq_(H) = 1.5  *U*_eq_(H) = 1.5 *U*_eq_(O) with C—H = 0.99 Å (CH_2_), C—H = 0.98 Å (CH_3_), O—H = 0.84 Å (OH), N—H = 0.93 Å (NH_1_) and N—H = 0.92 Å (NH_2_).

### Crystal data

**Table:**

[Cd_2_(NCS)_2_(SO_4_)(C_6_H_17_N_3_)_2_]·0.5CH_4_O	*Z* = 2
*M**_r_* = 715.49	*F*(000) = 718
Triclinic, *P*1	*D*_x_ = 1.738 Mg m^−^^3^
Hall symbol: -P 1	Mo *K*α radiation, λ = 0.71073 Å
*a* = 10.6648 (9) Å	Cell parameters from 13797 reflections
*b* = 12.4441 (12) Å	θ = 2.5–27.0°
*c* = 12.9240 (12) Å	µ = 1.82 mm^−^^1^
α = 61.359 (10)°	*T* = 200 K
β = 69.064 (10)°	Block, colourless
γ = 68.772 (10)°	0.18 × 0.13 × 0.09 mm
*V* = 1367.3 (2) Å^3^	

### Data collection

**Table:**

STOE IPDS-1 diffractometer	5736 independent reflections
Radiation source: fine-focus sealed tube	4365 reflections with *I* > 2σ(*I*)
graphite	*R*_int_ = 0.040
Phi scans	θ_max_ = 27.0°, θ_min_ = 2.5°
Absorption correction: numerical (*X-SHAPE* and *X-RED32*; Stoe & Cie, 2008)	*h* = −13→13
*T*_min_ = 0.746, *T*_max_ = 0.841	*k* = −15→15
12730 measured reflections	*l* = −16→16

### Refinement

**Table:**

Refinement on *F*^2^	Secondary atom site location: difference Fourier map
Least-squares matrix: full	Hydrogen site location: inferred from neighbouring sites
*R*[*F*^2^ > 2σ(*F*^2^)] = 0.042	H-atom parameters constrained
*wR*(*F*^2^) = 0.113	*w* = 1/[σ^2^(*F*_o_^2^) + (0.0508*P*)^2^ + 3.6107*P*] where *P* = (*F*_o_^2^ + 2*F*_c_^2^)/3
*S* = 1.07	(Δ/σ)_max_ = 0.001
5736 reflections	Δρ_max_ = 1.07 e Å^−^^3^
301 parameters	Δρ_min_ = −0.87 e Å^−^^3^
0 restraints	Extinction correction: *SHELXL97* (Sheldrick, 2008), Fc^*^=kFc[1+0.001xFc^2^λ^3^/sin(2θ)]^-1/4^
Primary atom site location: structure-invariant direct methods	Extinction coefficient: 0.0113 (8)

### Special details

**Table:**

Geometry. All esds (except the esd in the dihedral angle between two l.s. planes) are estimated using the full covariance matrix. The cell esds are taken into account individually in the estimation of esds in distances, angles and torsion angles; correlations between esds in cell parameters are only used when they are defined by crystal symmetry. An approximate (isotropic) treatment of cell esds is used for estimating esds involving l.s. planes.
Refinement. Refinement of F^2^ against ALL reflections. The weighted R-factor wR and goodness of fit S are based on F^2^, conventional R-factors R are based on F, with F set to zero for negative F^2^. The threshold expression of F^2^ > 2sigma(F^2^) is used only for calculating R-factors(gt) etc. and is not relevant to the choice of reflections for refinement. R-factors based on F^2^ are statistically about twice as large as those based on F, and R- factors based on ALL data will be even larger.

### Fractional atomic coordinates and isotropic or equivalent isotropic displacement parameters (Å^2^)

**Table:**

	*x*	*y*	*z*	*U*_iso_*/*U*_eq_	Occ. (<1)
Cd1	0.58718 (4)	0.60656 (4)	0.33218 (3)	0.02703 (13)	
Cd2	0.13789 (4)	0.35828 (4)	0.45817 (4)	0.03022 (14)	
N1	0.7929 (5)	0.6709 (5)	0.2127 (5)	0.0374 (12)	
C1	0.8391 (7)	0.7302 (7)	0.1166 (7)	0.0464 (16)	
S1	0.9043 (3)	0.8167 (3)	−0.0225 (2)	0.0857 (8)	
N2	0.3704 (6)	0.2779 (6)	0.4148 (6)	0.0497 (15)	
C2	0.4515 (6)	0.2311 (5)	0.3503 (6)	0.0344 (13)	
S2	0.5669 (2)	0.1683 (2)	0.2620 (2)	0.0638 (6)	
S3	0.23030 (13)	0.59733 (11)	0.45203 (11)	0.0225 (3)	
O1	0.3696 (4)	0.5152 (3)	0.4693 (3)	0.0237 (7)	
O2	0.1952 (4)	0.6886 (4)	0.5048 (4)	0.0341 (9)	
O3	0.1277 (4)	0.5182 (3)	0.5148 (3)	0.0248 (7)	
O4	0.2301 (4)	0.6606 (4)	0.3235 (3)	0.0358 (9)	
N11	0.4780 (5)	0.7650 (4)	0.3937 (4)	0.0314 (10)	
H11A	0.3903	0.7550	0.4365	0.038*	
H11B	0.5233	0.7579	0.4461	0.038*	
N12	0.4885 (5)	0.7225 (5)	0.1623 (4)	0.0335 (11)	
H12	0.3988	0.7116	0.1897	0.040*	
N13	0.6435 (5)	0.4318 (5)	0.2982 (4)	0.0328 (11)	
H13A	0.7145	0.3756	0.3353	0.039*	
H13B	0.5688	0.3957	0.3348	0.039*	
C11	0.4672 (7)	0.8939 (6)	0.2973 (6)	0.0451 (16)	
H11C	0.5611	0.9083	0.2545	0.054*	
H11D	0.4176	0.9555	0.3346	0.054*	
C12	0.3922 (7)	0.9159 (6)	0.2065 (6)	0.0490 (17)	
H12A	0.3086	0.8818	0.2515	0.059*	
H12B	0.3612	1.0079	0.1620	0.059*	
C13	0.4749 (7)	0.8584 (6)	0.1151 (6)	0.0457 (16)	
H13C	0.4304	0.9011	0.0455	0.055*	
H13D	0.5684	0.8747	0.0847	0.055*	
C14	0.5532 (7)	0.6788 (7)	0.0627 (5)	0.0433 (15)	
H14A	0.6449	0.6991	0.0227	0.052*	
H14B	0.4957	0.7254	0.0019	0.052*	
C15	0.5708 (8)	0.5377 (7)	0.1047 (6)	0.0468 (17)	
H15A	0.4827	0.5156	0.1590	0.056*	
H15B	0.5865	0.5210	0.0330	0.056*	
C16	0.6857 (8)	0.4500 (7)	0.1701 (6)	0.0454 (16)	
H16A	0.7104	0.3676	0.1652	0.054*	
H16B	0.7684	0.4857	0.1300	0.054*	
N21	0.1109 (6)	0.4989 (5)	0.2712 (4)	0.0381 (12)	
H21A	0.0189	0.5387	0.2760	0.046*	
H21B	0.1585	0.5590	0.2463	0.046*	
N22	0.0976 (5)	0.2022 (5)	0.4261 (5)	0.0375 (12)	
H22	0.0022	0.2148	0.4444	0.045*	
N23	0.0807 (5)	0.2394 (4)	0.6569 (4)	0.0324 (10)	
H23A	0.1097	0.2661	0.6978	0.039*	
H23B	−0.0140	0.2544	0.6804	0.039*	
C21	0.1564 (8)	0.4493 (7)	0.1779 (6)	0.0502 (17)	
H21C	0.2576	0.4155	0.1647	0.060*	
H21D	0.1359	0.5191	0.1010	0.060*	
C22	0.0864 (9)	0.3446 (8)	0.2112 (6)	0.057 (2)	
H22A	−0.0130	0.3704	0.2459	0.068*	
H22B	0.0938	0.3378	0.1358	0.068*	
C23	0.1440 (7)	0.2160 (7)	0.2992 (7)	0.0475 (17)	
H23C	0.1164	0.1521	0.2926	0.057*	
H23D	0.2461	0.1990	0.2762	0.057*	
C24	0.1475 (7)	0.0726 (6)	0.5072 (7)	0.0476 (17)	
H24A	0.2490	0.0549	0.4933	0.057*	
H24B	0.1265	0.0146	0.4869	0.057*	
C25	0.0848 (8)	0.0459 (6)	0.6396 (7)	0.0489 (17)	
H25A	0.1021	−0.0465	0.6857	0.059*	
H25B	−0.0164	0.0789	0.6499	0.059*	
C26	0.1370 (8)	0.1009 (6)	0.6962 (7)	0.0488 (17)	
H26A	0.1094	0.0595	0.7855	0.059*	
H26B	0.2391	0.0829	0.6728	0.059*	
O31	0.1929 (15)	0.7375 (11)	0.0583 (11)	0.076 (4)	0.50
H31	0.2769	0.7008	0.0475	0.114*	0.50
C31	0.1801 (14)	0.8574 (13)	0.0413 (12)	0.050 (4)	0.50
H31A	0.1703	0.8593	0.1188	0.075*	0.50
H31B	0.0983	0.9125	0.0087	0.075*	0.50
H31C	0.2624	0.8868	−0.0158	0.075*	0.50

### Atomic displacement parameters (Å^2^)

**Table:**

	*U*^11^	*U*^22^	*U*^33^	*U*^12^	*U*^13^	*U*^23^
Cd1	0.0291 (2)	0.0297 (2)	0.0207 (2)	−0.00729 (16)	−0.00580 (15)	−0.00850 (15)
Cd2	0.0294 (3)	0.0346 (2)	0.0326 (2)	−0.00777 (17)	−0.00505 (17)	−0.01921 (18)
N1	0.031 (3)	0.052 (3)	0.033 (3)	−0.020 (2)	−0.010 (2)	−0.010 (2)
C1	0.044 (4)	0.062 (4)	0.051 (4)	−0.018 (3)	−0.005 (3)	−0.036 (4)
S1	0.119 (2)	0.107 (2)	0.0407 (11)	−0.0754 (18)	0.0075 (12)	−0.0195 (12)
N2	0.029 (3)	0.070 (4)	0.072 (4)	−0.004 (3)	−0.010 (3)	−0.052 (4)
C2	0.030 (3)	0.033 (3)	0.047 (3)	−0.009 (2)	−0.013 (3)	−0.016 (3)
S2	0.0483 (11)	0.0642 (12)	0.0892 (15)	−0.0107 (9)	0.0066 (10)	−0.0557 (12)
S3	0.0163 (6)	0.0281 (6)	0.0226 (6)	−0.0081 (5)	−0.0038 (4)	−0.0080 (5)
O1	0.0176 (19)	0.0311 (18)	0.0237 (17)	−0.0043 (15)	−0.0064 (14)	−0.0117 (15)
O2	0.023 (2)	0.035 (2)	0.051 (2)	−0.0062 (17)	−0.0077 (17)	−0.0233 (19)
O3	0.0214 (19)	0.0316 (19)	0.0236 (18)	−0.0142 (15)	0.0006 (14)	−0.0111 (15)
O4	0.022 (2)	0.050 (2)	0.0214 (18)	−0.0120 (18)	−0.0080 (15)	0.0009 (17)
N11	0.036 (3)	0.031 (2)	0.033 (2)	−0.012 (2)	−0.007 (2)	−0.015 (2)
N12	0.030 (3)	0.044 (3)	0.018 (2)	−0.017 (2)	−0.0042 (18)	−0.0005 (19)
N13	0.034 (3)	0.040 (3)	0.031 (2)	−0.015 (2)	−0.002 (2)	−0.018 (2)
C11	0.046 (4)	0.027 (3)	0.055 (4)	−0.014 (3)	−0.006 (3)	−0.011 (3)
C12	0.037 (4)	0.034 (3)	0.048 (4)	−0.004 (3)	−0.008 (3)	0.001 (3)
C13	0.044 (4)	0.042 (3)	0.033 (3)	−0.017 (3)	−0.010 (3)	0.004 (3)
C14	0.041 (4)	0.068 (4)	0.020 (3)	−0.027 (3)	−0.002 (2)	−0.010 (3)
C15	0.050 (4)	0.076 (5)	0.030 (3)	−0.031 (4)	−0.002 (3)	−0.026 (3)
C16	0.053 (4)	0.059 (4)	0.035 (3)	−0.025 (3)	0.003 (3)	−0.028 (3)
N21	0.038 (3)	0.046 (3)	0.030 (3)	−0.011 (2)	−0.002 (2)	−0.018 (2)
N22	0.026 (3)	0.045 (3)	0.057 (3)	−0.005 (2)	−0.007 (2)	−0.036 (3)
N23	0.029 (3)	0.035 (3)	0.034 (3)	−0.007 (2)	−0.009 (2)	−0.013 (2)
C21	0.044 (4)	0.070 (5)	0.029 (3)	−0.009 (3)	0.004 (3)	−0.026 (3)
C22	0.059 (5)	0.089 (6)	0.042 (4)	−0.018 (4)	−0.010 (3)	−0.041 (4)
C23	0.038 (4)	0.063 (4)	0.064 (4)	−0.005 (3)	−0.011 (3)	−0.048 (4)
C24	0.043 (4)	0.038 (3)	0.076 (5)	−0.008 (3)	−0.015 (3)	−0.033 (3)
C25	0.047 (4)	0.030 (3)	0.070 (5)	−0.005 (3)	−0.017 (3)	−0.020 (3)
C26	0.055 (5)	0.033 (3)	0.052 (4)	−0.003 (3)	−0.025 (3)	−0.008 (3)
O31	0.101 (11)	0.064 (7)	0.055 (7)	−0.020 (7)	−0.039 (7)	−0.001 (6)
C31	0.034 (8)	0.058 (9)	0.039 (7)	−0.020 (6)	−0.027 (6)	0.014 (6)

### Geometric parameters (Å, °)

**Table:**

Cd1—N11	2.248 (4)	C14—C15	1.528 (10)
Cd1—N13	2.250 (5)	C14—H14A	0.9900
Cd1—N1	2.347 (5)	C14—H14B	0.9900
Cd1—N12	2.351 (5)	C15—C16	1.515 (11)
Cd1—O1^i^	2.388 (3)	C15—H15A	0.9900
Cd1—O1	2.619 (3)	C15—H15B	0.9900
Cd2—N23	2.247 (5)	C16—H16A	0.9900
Cd2—N21	2.250 (5)	C16—H16B	0.9900
Cd2—N2	2.283 (6)	N21—C21	1.466 (8)
Cd2—N22	2.374 (4)	N21—H21A	0.9200
Cd2—O3	2.386 (3)	N21—H21B	0.9200
Cd2—O3^ii^	2.676 (4)	N22—C24	1.463 (9)
N1—C1	1.130 (8)	N22—C23	1.475 (8)
C1—S1	1.633 (8)	N22—H22	0.9300
N2—C2	1.169 (8)	N23—C26	1.489 (8)
C2—S2	1.607 (6)	N23—H23A	0.9200
S3—O4	1.458 (4)	N23—H23B	0.9200
S3—O2	1.465 (4)	C21—C22	1.542 (11)
S3—O3	1.485 (3)	C21—H21C	0.9900
S3—O1	1.490 (4)	C21—H21D	0.9900
O1—Cd1^i^	2.388 (3)	C22—C23	1.508 (11)
N11—C11	1.480 (7)	C22—H22A	0.9900
N11—H11A	0.9200	C22—H22B	0.9900
N11—H11B	0.9200	C23—H23C	0.9900
N12—C13	1.466 (8)	C23—H23D	0.9900
N12—C14	1.480 (8)	C24—C25	1.510 (10)
N12—H12	0.9300	C24—H24A	0.9900
N13—C16	1.476 (7)	C24—H24B	0.9900
N13—H13A	0.9200	C25—C26	1.537 (10)
N13—H13B	0.9200	C25—H25A	0.9900
C11—C12	1.515 (10)	C25—H25B	0.9900
C11—H11C	0.9900	C26—H26A	0.9900
C11—H11D	0.9900	C26—H26B	0.9900
C12—C13	1.515 (10)	O31—C31	1.362 (18)
C12—H12A	0.9900	O31—H31	0.8400
C12—H12B	0.9900	C31—H31A	0.9800
C13—H13C	0.9900	C31—H31B	0.9800
C13—H13D	0.9900	C31—H31C	0.9800
			
N11—Cd1—N13	164.76 (17)	C15—C14—H14A	108.9
N11—Cd1—N1	99.40 (18)	N12—C14—H14B	108.9
N13—Cd1—N1	95.82 (18)	C15—C14—H14B	108.9
N11—Cd1—N12	89.43 (18)	H14A—C14—H14B	107.7
N13—Cd1—N12	89.82 (18)	C16—C15—C14	117.0 (5)
N1—Cd1—N12	89.72 (17)	C16—C15—H15A	108.0
N11—Cd1—O1^i^	85.80 (15)	C14—C15—H15A	108.0
N13—Cd1—O1^i^	91.31 (15)	C16—C15—H15B	108.0
N1—Cd1—O1^i^	104.15 (15)	C14—C15—H15B	108.0
N12—Cd1—O1^i^	165.89 (14)	H15A—C15—H15B	107.3
N11—Cd1—O1	84.77 (14)	N13—C16—C15	111.0 (5)
N13—Cd1—O1	80.07 (14)	N13—C16—H16A	109.4
N1—Cd1—O1	175.20 (16)	C15—C16—H16A	109.4
N12—Cd1—O1	92.73 (14)	N13—C16—H16B	109.4
O1^i^—Cd1—O1	73.63 (13)	C15—C16—H16B	109.4
N23—Cd2—N21	159.07 (19)	H16A—C16—H16B	108.0
N23—Cd2—N2	101.4 (2)	C21—N21—Cd2	116.5 (4)
N21—Cd2—N2	99.3 (2)	C21—N21—H21A	108.2
N23—Cd2—N22	86.47 (18)	Cd2—N21—H21A	108.2
N21—Cd2—N22	90.32 (19)	C21—N21—H21B	108.2
N2—Cd2—N22	90.40 (18)	Cd2—N21—H21B	108.2
N23—Cd2—O3	86.86 (14)	H21A—N21—H21B	107.3
N21—Cd2—O3	92.16 (15)	C24—N22—C23	110.7 (5)
N2—Cd2—O3	101.23 (16)	C24—N22—Cd2	114.7 (4)
N22—Cd2—O3	167.55 (15)	C23—N22—Cd2	114.1 (4)
C1—N1—Cd1	140.6 (5)	C24—N22—H22	105.4
N1—C1—S1	179.4 (9)	C23—N22—H22	105.4
C2—N2—Cd2	138.6 (5)	Cd2—N22—H22	105.4
N2—C2—S2	178.1 (6)	C26—N23—Cd2	116.8 (4)
O4—S3—O2	111.1 (3)	C26—N23—H23A	108.1
O4—S3—O3	108.7 (2)	Cd2—N23—H23A	108.1
O2—S3—O3	109.5 (2)	C26—N23—H23B	108.1
O4—S3—O1	109.9 (2)	Cd2—N23—H23B	108.1
O2—S3—O1	108.6 (2)	H23A—N23—H23B	107.3
O3—S3—O1	109.1 (2)	N21—C21—C22	113.1 (5)
S3—O1—Cd1^i^	120.41 (19)	N21—C21—H21C	109.0
S3—O1—Cd1	118.74 (19)	C22—C21—H21C	109.0
Cd1^i^—O1—Cd1	106.37 (13)	N21—C21—H21D	109.0
S3—O3—Cd2	122.13 (19)	C22—C21—H21D	109.0
C11—N11—Cd1	116.2 (4)	H21C—C21—H21D	107.8
C11—N11—H11A	108.2	C23—C22—C21	115.7 (6)
Cd1—N11—H11A	108.2	C23—C22—H22A	108.4
C11—N11—H11B	108.2	C21—C22—H22A	108.4
Cd1—N11—H11B	108.2	C23—C22—H22B	108.4
H11A—N11—H11B	107.4	C21—C22—H22B	108.4
C13—N12—C14	110.3 (5)	H22A—C22—H22B	107.4
C13—N12—Cd1	113.1 (4)	N22—C23—C22	113.7 (5)
C14—N12—Cd1	115.7 (4)	N22—C23—H23C	108.8
C13—N12—H12	105.6	C22—C23—H23C	108.8
C14—N12—H12	105.6	N22—C23—H23D	108.8
Cd1—N12—H12	105.6	C22—C23—H23D	108.8
C16—N13—Cd1	115.9 (4)	H23C—C23—H23D	107.7
C16—N13—H13A	108.3	N22—C24—C25	113.7 (5)
Cd1—N13—H13A	108.3	N22—C24—H24A	108.8
C16—N13—H13B	108.3	C25—C24—H24A	108.8
Cd1—N13—H13B	108.3	N22—C24—H24B	108.8
H13A—N13—H13B	107.4	C25—C24—H24B	108.8
N11—C11—C12	112.4 (5)	H24A—C24—H24B	107.7
N11—C11—H11C	109.1	C24—C25—C26	116.3 (6)
C12—C11—H11C	109.1	C24—C25—H25A	108.2
N11—C11—H11D	109.1	C26—C25—H25A	108.2
C12—C11—H11D	109.1	C24—C25—H25B	108.2
H11C—C11—H11D	107.8	C26—C25—H25B	108.2
C13—C12—C11	115.7 (6)	H25A—C25—H25B	107.4
C13—C12—H12A	108.4	N23—C26—C25	111.9 (6)
C11—C12—H12A	108.4	N23—C26—H26A	109.2
C13—C12—H12B	108.4	C25—C26—H26A	109.2
C11—C12—H12B	108.4	N23—C26—H26B	109.2
H12A—C12—H12B	107.4	C25—C26—H26B	109.2
N12—C13—C12	114.5 (5)	H26A—C26—H26B	107.9
N12—C13—H13C	108.6	C31—O31—H31	109.5
C12—C13—H13C	108.6	O31—C31—H31A	109.5
N12—C13—H13D	108.6	O31—C31—H31B	109.5
C12—C13—H13D	108.6	H31A—C31—H31B	109.5
H13C—C13—H13D	107.6	O31—C31—H31C	109.5
N12—C14—C15	113.4 (5)	H31A—C31—H31C	109.5
N12—C14—H14A	108.9	H31B—C31—H31C	109.5

Symmetry codes: (i) −*x*+1, −*y*+1, −*z*+1; (ii) −*x*, −*y*+1, −*z*+1.
